# Artificial Intelligence in Gastroenterology: Towards Explainable, Generalisable and Clinically Actionable Decision Support

**DOI:** 10.3390/diagnostics16193182

**Published:** 2026-09-30

**Authors:** İsmail Baydili, Burak Tasci, Sengul Dogan, Turker Tuncer

**Affiliations:** 1 Department of Audiovisual Techniques and Media Production, Vocational School of Technical Sciences, Firat University, Elazig 23119, Türkiye; 2 Vocational School of Technical Sciences, Firat University, Elazig 23119, Türkiye; 3 Department of Digital Forensics Engineering, Technology Faculty, Firat University, Elazig 23119, Türkiye; sdogan@firat.edu.tr (S.D.); turkertuncer@firat.edu.tr (T.T.)

**Keywords:** artificial intelligence, gastroenterology, explainable artificial intelligence, generalisability, clinical decision support, human–AI interaction, shared decision making, implementation science

## Abstract

Artificial intelligence is increasingly used in gastroenterology for endoscopic detection and characterisation, disease-activity assessment, risk prediction, treatment-response estimation and workflow support. However, high retrospective accuracy does not necessarily translate into reliable or beneficial clinical decision support. This structured narrative review examines the clinical development of artificial intelligence across gastrointestinal endoscopy, inflammatory bowel disease, hepatology and pancreaticobiliary diseases through three translational dimensions: explainability, generalisability and clinical actionability. Communication, human oversight and lifecycle governance are considered cross-cutting determinants of implementation. The evidence is most mature in gastrointestinal endoscopy, particularly for computer-aided polyp detection, although benefits vary across operators, indications and practice settings. Applications in inflammatory bowel disease, hepatology and pancreaticobiliary care remain predominantly retrospective, with limited prospective evidence that model outputs alter management or improve patient outcomes. Explainability is commonly represented by heatmaps or feature-attribution methods, but rarely evaluated in terms of clinician understanding, calibrated trust or patient communication. External validation is becoming more frequent, yet geographic and technological transportability, calibration and performance under dataset shift remain insufficiently assessed. Prospective implementation and health-economic studies are uncommon, and available economic evidence is largely model-based. Clinical artificial intelligence should therefore be evaluated as a sociotechnical intervention comprising the model, interface, users, workflow, communication process and associated care pathway. Translation requires clinically defined use cases, representative multicentre validation, human-centred evaluation, prospective assessment of benefits and harms, and continuous post-deployment surveillance. Clinical AI in gastroenterology should complement clinical expertise through transparent, contestable and continuously monitored decision support that preserves clinician accountability and supports informed, patient-centred decisions.

## 1. Introduction

Digestive diseases constitute a heterogeneous and substantial global health burden, encompassing common functional disorders, inflammatory and metabolic conditions, and gastrointestinal and hepatobiliary malignancies. An analysis of the Global Burden of Disease 2019 data estimated that digestive diseases accounted for approximately 8 million deaths and 277 million disability-adjusted life years worldwide in 2019, with marked differences across levels of socioeconomic development [1]. Their diagnosis and management increasingly require the combined interpretation of endoscopic images and videos, radiological and histopathological findings, laboratory measurements, electronic health records, and, in selected settings, molecular and multi-omics data. The scale and heterogeneity of these data, together with variability in clinical interpretation, have created a strong rationale for computational systems capable of supporting reproducible and timely patient-level decisions.

Artificial intelligence (AI), particularly machine learning and deep learning, has consequently been investigated across a broad range of gastroenterological applications [2]. Gastrointestinal endoscopy represents the most mature area, with computer-aided systems developed for lesion detection and characterisation, procedural quality assessment, automated reporting, and real-time guidance [3]. Beyond endoscopy, AI models have been used to assess endoscopic and histological activity in inflammatory bowel disease, predict relapse and treatment response, evaluate liver fibrosis and focal liver lesions, estimate prognosis in chronic liver disease and hepatocellular carcinoma, and support the diagnosis and risk stratification of pancreatic and biliary disorders [4,5]. Emerging multimodal approaches also aim to integrate imaging, clinical, histological, and molecular information to provide more individualised assessments than those based on a single data source [6].

Evidence of measurable clinical benefit is currently most developed in AI-assisted colonoscopy. A meta-analysis of 28 randomised controlled trials involving 23,861 participants found that computer-aided detection increased the adenoma detection rate by approximately 20% and reduced the adenoma miss rate [7]. However, the same analysis found no significant improvement in sessile serrated lesion detection and reported an increase in non-neoplastic resections, illustrating that improvements in a technical or intermediate endpoint do not necessarily translate into unequivocal clinical benefit.

Several multicentre studies explored the potential of transportability of AI systems. For instance, a real-time system for the detection of upper gastrointestinal cancer was evaluated using data from six hospitals, including prospective and external validation cohorts. In those cohorts, the sensitivity of cancer detection by the system was comparable to that of expert endoscopists [8]. Such studies, however, are less common than retrospective studies, which utilise curated data from a single institution.

Clinically useful AI requires outputs that can be interpreted in relation to uncertainty, failure modes and the decision at hand. Human–AI interaction therefore forms part of the clinical performance of the system, rather than a separate implementation consideration [9,10,11].

Clinical translation also depends on how uncertainty is communicated, how human oversight is maintained and whether performance remains stable across populations, institutions and workflows [12,13,14,15,16]. These cross-cutting issues are examined below alongside explainability, generalisability and clinical actionability. The contribution of this review is not to redefine explainability, generalisability or clinical actionability in isolation, but to examine their interdependence as conditions for translation. Explainability without transportable performance may rationalise unreliable predictions; generalisability without actionability may yield robust but clinically inert models; and actionability without interpretable uncertainty or external validity may amplify inappropriate decisions. We therefore treat these dimensions as a coupled translational framework, with communication, human oversight and lifecycle governance linking model performance to accountable clinical use.

## 2. Review Design and Methods

### 2.1. Review Approach

A focused, structured narrative review was conducted to synthesise clinically relevant evidence on AI-enabled decision support across heterogeneous study designs and application domains in gastroenterology. The review design was informed by established distinctions among evidence-review methodologies and by the SANRA framework for narrative review articles. Study selection was purposive, with emphasis on methodological robustness, clinical relevance and contribution to the review’s translational themes [17,18]. The objective was not to produce an exhaustive estimate of intervention effects, but to examine why promising technical performance does or does not translate into explainable, generalisable and clinically actionable decision support.

The review encompassed gastrointestinal endoscopy, inflammatory bowel disease, hepatology, pancreaticobiliary disorders, gastrointestinal oncology and pathology, and general gastroenterology practice. Disease-specific examples were selected to illustrate recurrent translational challenges across contrasting clinical settings rather than to provide exhaustive coverage of each subspecialty. This structure was intended to support comparison across domains while maintaining focus on explainability, generalisability and clinical actionability. Health communication was treated as a cross-cutting mechanism through which AI outputs influence clinician judgement, patient understanding, trust, accountability and shared decision making.

### 2.2. Evidence Identification

Web of Science Core Collection, Scopus and PubMed were searched using complementary search sets designed to identify evidence concerning: broad clinical applications of AI in gastroenterology; explainability and interpretability; external validity and generalisability; clinical utility and implementation; the burden of digestive diseases; clinician–AI interaction; and patient communication and shared decision making.

The searches combined terms describing AI, machine learning, deep learning, neural networks, computer-aided detection or diagnosis, and large language models with terms for gastroenterology, gastrointestinal diseases, endoscopy, hepatology, inflammatory bowel disease and pancreaticobiliary disorders. Additional concepts included clinical decision support, implementation, external validation, explainability, workflow, trust, usability, health literacy, risk communication, informed consent and shared decision making.

The principal clinical searches covered publications from 2018 to 2026. Searches intended to identify recent field-level reviews were restricted to 2021–2026, whereas the digestive-disease burden search covered 2020–2026. English-language articles and reviews were considered. Professional guidance, consensus statements and position papers indexed within these categories were retained when they directly addressed the clinical implementation or governance of AI.

Records retrieved from Web of Science Core Collection, Scopus, and PubMed were combined and deduplicated hierarchically using DOI and database-specific identifiers, including the Web of Science accession number, Scopus EID, and PubMed ID (PMID), where available. Normalised full-title matching was used when persistent or database-specific identifiers were unavailable.

### 2.3. Eligibility and Study Prioritisation

Primary studies were considered relevant when AI was a substantive component of a clinical or communication-related task in gastroenterology. Eligible tasks included detection, diagnosis, prognostication, treatment selection, disease monitoring, procedural support, workflow optimisation, patient education and clinician–patient communication. Reviews and professional guidance were retained when they provided clinically relevant synthesis, implementation recommendations or governance frameworks.

Studies addressing clinician or patient trust, usability, cognitive workload, automation bias, algorithm aversion, uncertainty communication, health literacy, informed consent, professional responsibility or shared decision making were eligible when these issues were measured or substantively analysed. Statements presenting communication or trust merely as a possible future benefit, without empirical evaluation or developed analysis, were not considered communication evidence.

We excluded studies in which gastrointestinal terminology was incidental, the main clinical context was outside gastroenterology, or AI was mentioned only as a prospective opportunity. Animal studies, preclinical experiments and drug-discovery studies were excluded. Molecular, genomic, omics and radiomics studies were considered only when they addressed a defined patient-level clinical decision or directly informed one of the review’s translational dimensions. Technical model-development studies were prioritised only when they informed at least one of the three principal dimensions or were linked to a recognisable clinical pathway. Protocols without completed results and meeting abstracts lacking sufficient methodological information were not treated as clinical evidence.

Titles and abstracts were assessed against the prespecified eligibility criteria, and uncertain or borderline records were reviewed before inclusion in the narrative synthesis. Study prioritisation considered clinical relevance, methodological strength and proximity to practice, with greater weight given to prospective, multicentre, externally validated and human–AI studies reporting clinically meaningful comparators or outcomes. Both favourable and attenuated or negative findings were retained when they informed the assessment of clinical translation. The five core searches yielded, records in aggregate. After hierarchical deduplication, 1859 unique records remained, of which 1581 were provisionally retained and 278 excluded during title-and-abstract screening.

### 2.4. Evidence Characterisation

For prioritised studies, we considered the clinical domain, AI technology and task, data modality, study design, setting, number of centres, sample size, comparator, validation strategy and reported clinical outcome. Evidence was then organised around the review’s three principal dimensions.

Explainability was distinguished between technical attribution methods such as saliency maps, attention mechanisms or feature-importance analyses and explanations whose usefulness was evaluated with clinicians or patients. Generalisability was assessed with reference to the separation between development and evaluation data, temporal or external validation, institutional and geographical diversity, device or platform variation, and prospective or real-world testing. Clinical actionability was considered progressively, from model discrimination alone to changes in clinician performance, decision making, workflow, resource use or patient outcomes.

The communication analysis considered the intended audience, presentation of predictions and uncertainty, clinician–AI interaction, trust calibration, usability, automation bias, algorithm aversion, patient education, health literacy, informed consent, shared decision making and professional accountability. Studies could contribute to more than one analytical dimension.

### 2.5. Narrative Synthesis

Evidence was synthesised thematically rather than pooled statistically. Findings were interpreted according to study design, validation context and proximity to clinical practice. Prospective, multicentre, randomised and real-world studies were accorded greater interpretive weight than retrospective model-development studies, cross-sectional attitude surveys or expert opinion. Citation counts were used only to support navigation of the literature and were not treated as indicators of methodological quality. Empirical findings, guidance-based recommendations and interpretive conclusions were distinguished according to their underlying evidential basis.

Meta-analysis was not undertaken because of substantial heterogeneity in clinical tasks, populations, AI architectures, comparators and outcome definitions. Given the heterogeneity of study designs, no single risk-of-bias instrument was applied across the corpus. Evidence was instead interpreted according to study design, validation context, comparator quality, human evaluation and proximity to clinical practice.

### 2.6. Gastrointestinal Endoscopy: A Mature but Context-Dependent Evidence Base

Gastrointestinal endoscopy has the most developed interventional evidence base for AI in gastroenterology. This position reflects a favourable combination of standardised image acquisition, clearly defined procedural tasks and outcomes that can be measured during routine care. Computer-aided detection (CADe) has consequently progressed from retrospective image classification to real-time, multicentre randomised trials. Computer-aided diagnosis (CADx), automated quality monitoring and report generation have followed, although their readiness for clinical deployment remains less certain.

The strongest evidence concerns CADe during colonoscopy. In the pragmatic COLO-DETECT trial, conducted across 12 National Health Service hospitals in England, AI-assisted colonoscopy increased the mean number of adenomas detected per procedure from 1.21 to 1.56 and the adenoma detection rate from 48.4% to 56.6% [19]. A separate six-centre trial involving 3059 asymptomatic participants reported a similarly favourable effect: the adenoma detection rate increased from 32.4% with conventional colonoscopy to 39.9% with AI assistance, with improvements observed among both expert and non-expert endoscopists [20]. These trials show that CADe can improve lesion recognition beyond retrospective test sets and under clinically realistic conditions.

The benefit is not, however, consistent across populations or endpoints. In a Spanish colorectal cancer screening programme, CADe increased the detection of some small, non-polypoid and proximal lesions but did not improve the detection of advanced colorectal neoplasia, which was identified in 34.8% of AI-assisted procedures and 34.6% of controls [21]. Similarly, the international TIMELY trial found no increase in adenomas per colonoscopy among individuals with Lynch syndrome: the mean was 0.64 in both the AI-assisted and white-light endoscopy groups [22]. High baseline performance, careful mucosal inspection and the specialised nature of surveillance in Lynch syndrome might have reduced the marginal benefit of automated detection. Collectively, these results indicate that an increase in the number of detected lesions cannot be assumed to translate into improved detection of biologically consequential disease.

Evidence from community practice further illustrates the importance of setting. In the GAIN trial, the DEEP2 system increased adenomas per colonoscopy from 0.60 to 0.74 [23]. The overall difference in adenoma detection rate did not meet the prespecified significance threshold, however, and the apparent benefit within community clinics arose from a post hoc analysis. The finding is therefore hypothesis-generating rather than definitive. Nevertheless, it raises the possibility that CADe may offer greater value in settings with more variable baseline performance than in highly controlled expert centres. This potential interaction between technology, operator experience and local workflow is central to assessing generalisability.

The transition from detection to diagnosis is more difficult. In a prospective multicentre evaluation of real-time CADx for colorectal polyp histology, experienced endoscopists achieved greater accuracy than the automated system (75.2% versus 71.6%) and greater sensitivity for neoplastic polyps [24]. Agreement between the endoscopist and CADx increased accuracy to 78.1%, suggesting that concordant human–AI assessment may be more useful than either output considered in isolation. Confidence thresholds, error asymmetry and the consequences of an incorrect surveillance recommendation remain clinically decisive. A 2024 systematic review and meta-analysis of 11 studies comprising 7400 diminutive polyps further evaluated CADx within a resect-and-discard strategy. CADx assistance did not increase the proportion of polyps diagnosed with high confidence, but modestly reduced incorrect optical predictions compared with unassisted diagnosis (risk ratio 0.88, 95% CI 0.79–0.98); the certainty of evidence was low. These findings suggest that CADx may improve diagnostic consistency, but do not yet establish sufficiently robust evidence for routine pathology-sparing implementation [25].

Upper gastrointestinal endoscopy presents an equally mixed picture. A single-centre tandem randomised trial reported that AI assistance reduced the gastric-neoplasm miss rate from 27.3% to 6.1% [26]. By contrast, a subsequent trial involving 29,514 participants across 24 hospitals found no significant improvement in pathology-confirmed gastric-neoplasm detection (1.42% with AI assistance versus 1.25% without it). AI reduced the number of blind spots but also prolonged inspection and procedure times [27]. In oesophageal screening, a 12-centre tandem trial found numerically lower miss rates for superficial squamous-cell carcinoma and precancerous lesions, but the difference was not statistically conclusive [28]. These findings show how effects observed in single-centre or enriched settings can attenuate when an intervention is tested across institutions, operators and lower-prevalence populations.

Endoscopy therefore illustrates both the progress and the limitations of clinical AI. Randomised evidence supports the ability of CADe to increase detection in selected colonoscopy settings, but improvements are concentrated in surrogate procedural endpoints such as adenoma detection rate, adenomas per colonoscopy and lesion miss rate. Evidence that these systems reduce interval cancer, disease-specific mortality or other patient-important outcomes remains limited. CADx is less mature, and the available studies do not support unqualified diagnostic autonomy.

The central question has consequently shifted. It is no longer simply whether AI can detect additional lesions, but when those detections are clinically important, in which settings they remain reproducible, and how clinicians should interpret and act on the output. Alert timing, false-positive burden, confidence communication and the clinician’s response to agreement or disagreement with the system are part of the intervention itself. Endoscopy thus provides the clearest example of why technical performance must be considered together with explainability, generalisability, clinical actionability and human–AI communication.

### 2.7. Inflammatory Bowel Disease: From Automated Assessment to Treatment Decisions

The role of AI in inflammatory bowel disease (IBD) differs fundamentally from its use in lesion detection. The relevant decisions are longitudinal: whether inflammation is adequately controlled, whether an apparently quiescent disease will relapse, and which treatment is most likely to produce durable remission. AI could support these decisions by standardising endoscopic assessment and integrating clinical, laboratory, imaging and molecular data. The evidence, however, remains uneven. Automated assessment of ulcerative colitis is relatively mature, whereas treatment selection and outcome prediction remain largely investigational.

Endoscopic scoring is the best-developed application. In a single-centre diagnostic study involving 16,514 colonoscopy images from 3082 patients with ulcerative colitis, a convolutional neural network distinguished endoscopic remission from moderate-to-severe disease with an area under the receiver operating characteristic curve (AUROC) of 0.966. Agreement with the adjudicated Mayo endoscopic subscore was similar to that between experienced reviewers, and performance remained high when the model was applied to 30 previously unseen colonoscopy videos [29]. Although the video analysis moved beyond isolated images, it did not constitute geographically independent validation.

A deep neural network subsequently trained on more than 40,000 images and 6885 biopsy results identified endoscopic remission with 90.1% accuracy and histological remission with 92.9% accuracy in a prospectively enrolled, temporally separate cohort [30]. This study showed that automated assessment could extend beyond visual severity grading to estimation of histological healing. Nevertheless, development and validation occurred within the same Japanese centre, and the reference standard for endoscopic remission remained the assessment reported by endoscopists. The findings therefore support reproducibility over time more strongly than transportability across institutions or populations.

Multicentre testing has exposed a measurable generalisation gap. The vision-transformer-based UC-former achieved 90.8% accuracy on its internal test set, but accuracy declined to 82.4–85.0% across three external hospitals [31]. This reduction does not negate the value of the model; rather, it shows why internal performance cannot be assumed to persist across acquisition protocols, endoscopy systems and case mixtures. More recent work has begun to examine how AI affects the clinician rather than merely comparing their respective scores. In a retrospective multicentre study, an explainable model improved Mayo score classification by both a senior and a junior endoscopist, with accuracy increasing from 0.890 to 0.930 and from 0.810 to 0.880, respectively [32]. These results provide an early indication that explanation and human–AI interaction may improve interpretation, although the study did not evaluate whether this changed treatment or patient outcomes.

Prospective evidence connecting automated endoscopy with subsequent disease course is emerging. In a single-centre cohort of patients with ulcerative colitis in clinical remission, real-time AI-assisted colonoscopy classified mucosa as healing or active. Twelve-month relapse occurred in 28.4% of patients classified as AI-active, compared with 4.9% of those classified as AI-healing [33].The study demonstrates prognostic stratification during a live procedure, but it was observational: clinicians were not randomised to act or not act on the AI output. It therefore remains unknown whether AI-guided treatment adjustment reduces relapse, avoids unnecessary escalation or improves resource use.

Treatment-response prediction presents a more demanding test of clinical utility. The EPIC-CD study developed peripheral-blood DNA methylation signatures to predict response to adalimumab, vedolizumab and ustekinumab in Crohn’s disease [34]. Apparent discrimination was high in the discovery cohort, with AUROCs of 0.86–0.89. In the independent Oxford cohort, however, the adalimumab model failed markedly, with an AUROC of 0.25, whereas the vedolizumab and ustekinumab models achieved AUROCs of 0.75. Previous anti-tumour necrosis factor exposure further reduced performance. EPIC-CD therefore provides a particularly informative example of treatment-specific dataset shift: a biological signature that appears predictive during development might fail when therapeutic history and population characteristics change.

A prospective multi-omics model for response to exclusive enteral nutrition in paediatric Crohn’s disease showed more favourable external performance [35]. Integration of serum and faecal metabolites, lipid profiles and microbiome features produced an AUROC of 0.81 in an independent validation cohort. The cohort comprised only 21 participants, however, resulting in a wide confidence interval of 0.60–1.00. Such models illustrate the promise of biologically informed prediction but also introduce substantial practical requirements concerning specimen processing, assay harmonisation, cost and reproducibility. Their clinical value cannot be inferred from discrimination alone.

Negative validation results are equally instructive. When a previously developed random-forest algorithm was applied to laboratory data from 240 participants in the multicentre TOPPIC trial, it discriminated postoperative clinical recurrence of Crohn’s disease poorly, with an AUROC of 0.62 [36]. Performance for selected endoscopic and biochemical outcomes was modestly higher, but remained insufficient to support treatment decisions. The study shows that access to high-quality clinical-trial data does not ensure that a model developed for one therapeutic context will transfer to another.

IBD thus reveals a widening distance between automated measurement and decision support. AI can reproduce endoscopic activity scores, identify remission and stratify relapse risk with increasing technical sophistication. Evidence is nevertheless concentrated in ulcerative colitis imaging, while Crohn’s disease prediction frequently depends on smaller, heterogeneous clinical or multi-omics cohorts. Calibration is not routinely reported, independent validation often reduces performance, and no compelling prospective evidence yet shows that an AI-guided treatment strategy improves patient outcomes.

Clinical translation will require more than increasingly complex predictors. Models must produce calibrated, treatment-specific risk estimates, identify the population to which those estimates apply and communicate uncertainty in a form that clinicians and patients can use. In IBD, where alternative treatments have different safety profiles, burdens and patient preferences, actionability ultimately depends on whether predictions can support not displace deliberation and shared decision making.

### 2.8. Hepatology: From Risk Stratification to Consequential Clinical Decisions

In hepatology, artificial intelligence is being developed less as a real-time perceptual aid than as a means of integrating longitudinal, imaging, laboratory, and pathological data. The intended decisions are correspondingly consequential: whether to perform biopsy, intensify surveillance, anticipate liver-related events, select systemic treatment, or allocate patients to resection or transplantation. Although several models have now progressed beyond internal validation, evidence that their use improves clinical decisions or patient outcomes remains limited.

AI-assisted pathology provides one of the more mature examples. In a multisite validation involving 1481 biopsies from four completed MASH trials, AI-assisted expert assessment improved the accuracy of scoring inflammation, hepatocellular ballooning, composite disease activity, and MASH resolution, while maintaining non-inferiority for steatosis and fibrosis. Inter-reader variability was also reduced [37]. This is an important demonstration of human–AI complementarity, but its immediate application is the standardisation of clinical-trial eligibility and histological endpoints rather than autonomous diagnosis in routine care. Non-invasive models may have wider clinical reach. In 1198 patients with chronic hepatitis B and hepatic steatosis recruited across ten centres, machine-learning models based on routine clinical variables showed externally validated discrimination for advanced fibrosis and cirrhosis and outperformed established non-invasive indices [38]. Nevertheless, improved discrimination alone does not establish whether the model can safely reduce biopsy, alter treatment initiation, or improve downstream outcomes.

Longitudinal risk prediction may provide a more direct route to clinical action. The AI-Safe-C score was developed in 913 Korean patients without cirrhosis after sustained virological response and externally evaluated in 1264 patients from Hong Kong and France. The model achieved a C-index of 0.86 for liver-related events and distinguished a subgroup whose five-year event risk approached that observed in advanced fibrosis [39]. Such stratification could support risk-adapted follow-up after hepatitis C cure. However, the safety of intensifying surveillance in high-risk patients or reducing it in those classified as low-risk has not been tested prospectively.

Similar considerations apply to HCC surveillance. A gradient-boosting model developed in antiviral-treated chronic hepatitis B was externally validated in independent Korean and predominantly Caucasian cohorts, comprising 13,508 patients overall. Discrimination was preserved across populations, and a minimal-risk subgroup had an HCC incidence below 0.5% over eight years [40]. The study therefore addressed an important dimension of transportability, although the clinical consequences of surveillance de-intensification remain uncertain. Imaging-based systems may identify risk before a lesion becomes clinically apparent. The ALARM model, which combined three-phase CT radiomics, deep-learning features, and clinical variables, was evaluated in 1858 patients with cirrhosis across 11 centres. It achieved an AUC of 0.918 in the external cohort for HCC developing within the subsequent 3–12 months [41]. Yet its retrospective design, dependence on multiphase CT, and potential sensitivity to acquisition protocols constrain immediate implementation in population surveillance.

The field is also moving from prognosis towards treatment selection. An AI pathology model derived an atezolizumab–bevacizumab response signature directly from HCC histology and generalised across independent resection and biopsy cohorts. Among 122 treated patients, a high predicted signature was associated with longer median progression-free survival than a low signature 12 versus 7 months [42]. More recently, a model based on routinely collected clinical variables was developed using 774 patients treated with atezolizumab–bevacizumab across 24 centres in eight countries. External discrimination was stronger for overall survival than for progression-free survival, with AUROCs of 0.75 and 0.64, respectively; the latter did not consistently exceed established clinical predictors [43]. These findings illustrate both the potential and the limitations of AI-derived treatment stratification: association with outcomes after treatment should not be interpreted as evidence that the model identifies differential treatment benefit without an appropriate comparator or prospective treatment-allocation study.

The clearest attempt to connect prediction with a clinical decision examined selection between liver transplantation and surgical resection. Separate machine-learning models were developed in a nationwide cohort of 3915 patients and tested in an independent cohort of 614 patients. Counterfactual analyses suggested better survival under model-recommended treatment, but this benefit was simulated from retrospective data rather than observed after model-guided care [44]. Prospective evaluation is therefore essential before such recommendations influence access to a scarce and potentially life-saving resource.

Collectively, the hepatology literature shows progress from technical model development towards multicentre validation and decision-oriented outputs. Nevertheless, external validation should not be conflated with clinical actionability. Translation will require temporally and geographically representative testing, calibration across liver disease aetiologies, and predefined thresholds linked to explicit clinical actions. Communication is particularly important when model outputs could reduce surveillance, influence transplant eligibility, or shape systemic therapy. Clinicians and multidisciplinary teams need more than a risk category or feature-attribution plot: they need calibrated uncertainty, the factors driving the recommendation, and a clear account of what clinical action if any the prediction justifies. Patients, in turn, must be able to understand that a model-informed estimate modifies rather than determines an individual decision.

### 2.9. Pancreaticobiliary Endoscopy and Acute Pancreatitis: From Lesion Characterisation to Risk-Guided Management

The relevance of pancreaticobiliary AI to gastroenterology rests on its connection to endoscopic and medical decisions. Accordingly, the evidence considered here is restricted to EUS-based lesion characterisation, cholangioscopic assessment of biliary strictures, post-ERCP risk estimation, and acute pancreatitis management. Within this pancreaticobiliary subsection, CT-only radiomics, surgical planning, postoperative prognosis, molecular biomarker discovery and standalone digital pathology were not considered as primary clinical evidence.

EUS provides a natural setting for AI assistance because image interpretation and tissue acquisition remain operator-dependent. CH-EUS MASTER combined real-time lesion segmentation with benign–malignant classification and was evaluated prospectively in patients undergoing contrast-enhanced harmonic EUS. The system achieved 93.8% diagnostic accuracy and outperformed participating endoscopists for pancreatic mass classification. In the randomised procedural component, AI guidance increased the first-pass diagnostic yield of EUS-guided fine-needle aspiration, although several other sampling outcomes did not differ significantly between groups [45]. This distinction is important: improved image classification does not necessarily translate into a clinically meaningful improvement across the full sampling pathway.

Broader datasets have produced high technical performance but weaker evidence of transportability. A CNN developed from 378 EUS examinations and approximately 126,000 images obtained at four international centres distinguished common cystic and solid pancreatic lesions with reported accuracies of 94–99% [46]. However, examinations were pooled before a 90:10 training–test split, meaning that multicentre development should not be interpreted as independent external validation. Similarly, a retrospective model developed from 22,000 images in 933 patients achieved an AUC of 0.90 for differentiating pancreatic carcinomas from non-carcinomatous masses, but lacked external validation and showed uneven sensitivity across uncommon lesion types [47]. These studies demonstrate technical feasibility while also showing how image-level sample size can obscure the smaller number of independent patients and clinical sites.

AI-assisted endomicroscopy offers a more specific route to risk stratification in pancreatic cysts. In 63 surgically resected branch-duct IPMNs, an nCLE-based algorithm detected high-grade dysplasia or invasive cancer with greater accuracy in non-gastric than gastric subtypes [48]. The study used blinded pathological reclassification and consensus review, strengthening the reference standard. Nevertheless, the small, surgically enriched cohort limits extension to the broader surveillance population, in whom the central question is whether AI can safely avoid surgery without missing advanced neoplasia.

Biliary endoscopy has begun to generate stronger external validation evidence. A previously developed cholangioscopy AI system was tested without retraining across three academic centres. Among 61 examinations performed for biliary strictures, the system achieved 80.0% sensitivity, 90.3% specificity, and 85.2% accuracy for malignancy, significantly exceeding brush cytology, forceps biopsy, and their combination [49]. A subsequent transcontinental study included 135 digital single-operator cholangioscopy examinations from 129 patients across 14 centres in six countries. Patient-level classification achieved an AUC of 0.904, with 84.1% sensitivity and 85.7% specificity, and performance was reportedly maintained across centres and demographic groups [50]. This geographical and device diversity is encouraging, but the analysis used selected clinically relevant frames rather than uninterrupted real-time video. The effect of AI assistance on endoscopist decisions, tissue acquisition, repeat procedures, and time to diagnosis therefore remains uncertain.

Risk prediction is closer to clinical action when it informs a defined intervention. A gradient-boosting calculator for post-ERCP pancreatitis was developed from individual-level data for 7389 participants enrolled in 12 randomised trials. Its discrimination was moderate in development and prospective pilot cohorts with AUCs of 0.70 and 0.74, respectively, with a high negative predictive value but a positive predictive value of only 15% [51]. The calculator may help identify low-risk patients and support prophylaxis or monitoring decisions; however, the underlying trials evaluated prophylactic interventions, not allocation by the AI tool. Clinical benefit from model-guided prophylaxis therefore remains unproven.

A more directly management-oriented example is ADAPT, which generates dynamic fluid recommendations for acute pancreatitis. In a prospective cohort of 1083 patients, fluid administration exceeding the model recommendation was associated with respiratory failure, intensive-care admission, persistent systemic inflammation, longer hospitalisation, and local complications [52]. These findings connect model output with clinically important outcomes, but the comparison was observational rather than randomised. Adherence to the recommendation may therefore mark differences in baseline severity or clinical management that statistical adjustment cannot fully remove.

Overall, pancreaticobiliary AI is moving from retrospective image classification towards externally validated endoscopic systems and intervention-linked risk estimates. Its clinical maturity nevertheless varies sharply by use case. The most credible systems operate at a recognisable decision point: where to sample, whether a stricture is malignant, which prophylaxis to use, or how much fluid to administer. Future evaluation should use patient-level separation, continuous video, prespecified action thresholds, calibration at local disease prevalence, and prospective comparisons of AI-guided versus usual care. Because these decisions may lead to biopsy, surgery, repeat ERCP, intensive monitoring, or reduced surveillance, outputs must also communicate uncertainty clearly to both clinicians and patients rather than presenting a categorical prediction as a definitive diagnosis.

### 2.10. Explainability: From Visual Attribution to Clinically Meaningful Decision Support

Explainability is often treated as an intrinsic property of an artificial intelligence model, although its clinical value depends on what is explained, to whom, and for which decision. In gastroenterology, existing approaches can be considered at three levels. Technical interpretability uses saliency maps, attention maps, feature-importance scores, or segmentation overlays to indicate which inputs influenced a prediction. Clinically grounded explainability translates these signals into recognisable findings, such as mucosal irregularity, pit pattern, ulcer morphology, glandular architecture, or variceal features. Human-centred explainability goes further by testing whether the intended user understands the explanation, appropriately calibrates trust, and makes a safer or more accurate decision. Only this final level establishes that an explanation has clinical utility rather than merely visual plausibility.

Most published systems remain at the first two levels. Recent gastric histopathology work has used Grad-CAM visualisation to support interpretable classification, illustrating the continued use of attribution-based methods in technically oriented diagnostic systems; however, the clinical usefulness of these explanations was not evaluated with intended users [53]. In computational pathology, the IGUANA graph-learning model combined heatmaps with quantitative histological features and maintained an area under the receiver operating characteristic curve of approximately 0.97 across three external datasets. At a threshold selected for 99% sensitivity, the model was estimated to reduce the number of normal large-bowel biopsy slides requiring review by approximately 55% [54]. Similarly, an ulcerative colitis histology system represented predictions through tissue and cellular features and showed close agreement with the Nancy Histological Index [55]. These outputs are more clinically legible than an unqualified probability score, but neither study established whether the explanations improved pathologists’ decisions, prevented errors, or reduced workload in routine practice.

Endoscopic systems have increasingly incorporated domain knowledge into their outputs. A cholangioscopy model linked predictions of malignant biliary strictures to four recognised endoscopic features and detected 92.3% of malignant strictures in prospective video testing [56]. ENDOANGEL-GEV provided segmentation, variceal grading and red-colour sign classification, and its recommendations suggested that more patients might receive appropriate prophylactic intervention than under unaided endoscopic assessment [57]. However, concordance between an algorithm’s output and familiar clinical features does not, by itself, demonstrate that those features faithfully explain the model’s reasoning. Nor does it show that clinicians can recognise when an apparently coherent explanation accompanies an incorrect prediction.

Semantic explanations may provide a more useful bridge between model output and clinical reasoning, but they require validation as predictions in their own right. An explainable system for *Helicobacter pylori* infection generated nine mucosal features and achieved an external diagnostic accuracy of 91.9%; nevertheless, the proposed improvement in clinician trust was inferred rather than empirically measured [58]. In a colorectal polyp study, automatically generated textual descriptions agreed well with expert reference descriptions for some characteristics, including pit type, but poorly for others, particularly pit distribution. Agreement coefficients ranged from 0.167 to 0.957 across individual features [59]. This variation shows that a model can provide a fluent or clinically familiar explanation while mischaracterising the feature that the clinician is expected to use. Explanation accuracy should therefore be reported separately from diagnostic accuracy.

A smaller group of studies has evaluated explanations within human–AI interaction. ENDOANGEL-LA used predefined endoscopic features to explain predictions of early gastric cancer; assistance improved novice performance and was associated with greater user satisfaction than a conventional black-box model [60]. ENDOANGEL-ED subsequently evaluated 77 endoscopists and increased their diagnostic accuracy from 70.61% to 79.63%. Explainable output also produced higher ratings of trust and acceptance than the corresponding conventional deep-learning system [9]. In a multicentre randomised crossover study of pancreatic lesions, multimodal AI increased novice endoscopists’ accuracy from 0.69 to 0.90, while supplementary interpretability information reduced scepticism among experienced endoscopists [61]. More recently, an interpretable system that localised and graded Crohn’s disease ulcers improved the sensitivity and grading consistency of less-experienced readers [62]. These studies provide stronger evidence than technical visualisation alone because explanations were evaluated in relation to their intended users. Nonetheless, most measured short-term reader performance, satisfaction, or stated trust under controlled conditions. Whether these effects persist in routine care, improve patient outcomes, or instead encourage inappropriate reliance remains uncertain.

Clinically useful explainability should therefore be designed as a communication function rather than as a decorative layer placed over a prediction. A clinician-facing explanation should identify the evidence supporting the output, communicate uncertainty, disclose relevant limitations, and clarify how the result relates to the immediate decision. Different users will require different forms of explanation: an endoscopist may need lesion localisation and competing diagnostic features; a pathologist may require traceable morphological evidence; and a patient needs a plain-language account of what the result means, how certain it is, and whether it changes the available options. Patient-facing communication should also distinguish clearly between an algorithmic estimate and the clinician’s final recommendation, particularly when AI informs surveillance intervals, biopsy decisions, cancer risk, or treatment selection.

**Figure 1:**
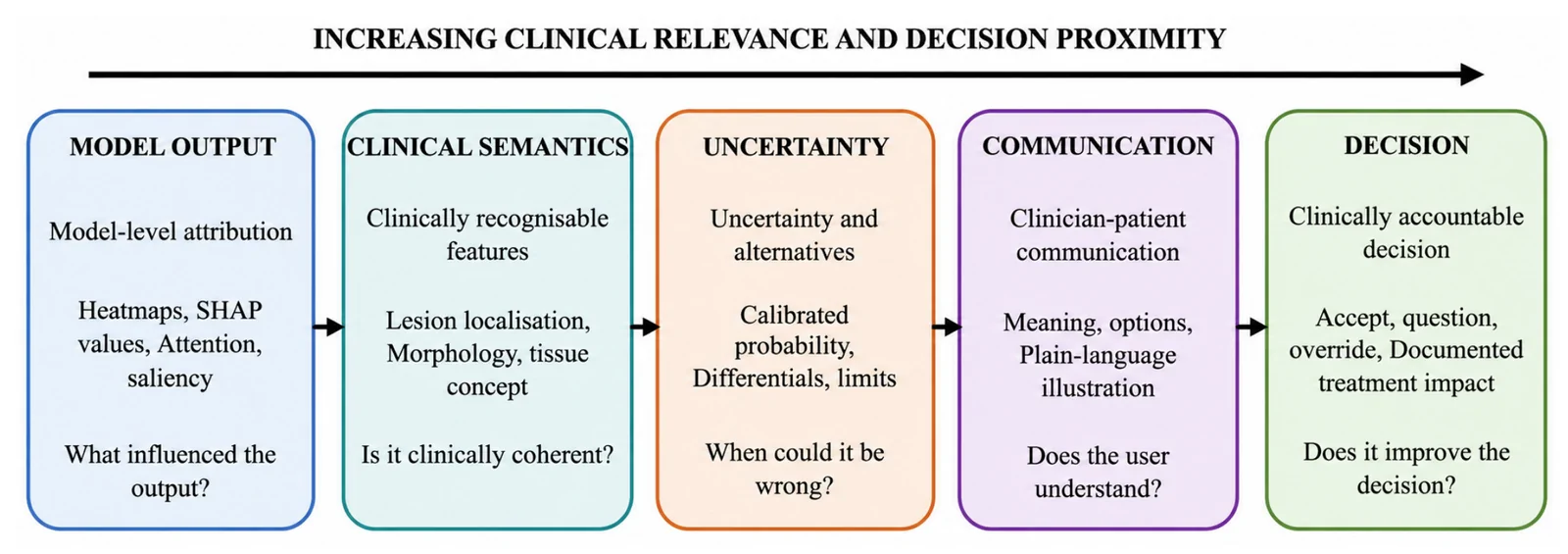
From technical attribution to clinically actionable explanation. Model-level attribution methods identify inputs associated with a prediction but do not establish clinical usefulness. Clinically meaningful explainability requires recognisable features, communication of uncertainty and support for accountable clinical decisions.

Future evaluation should consequently examine explanation fidelity, clinical coherence, user comprehension, trust calibration, decision quality and safety during clinician–AI disagreement. Prospective studies should compare prediction alone with prediction plus explanation and prediction plus explanation and uncertainty, while documenting overrides, automation bias and subgroup-specific failures. Explainability becomes clinically actionable only when it helps clinicians and patients understand when an AI recommendation should be accepted, questioned, or disregarded. Explainability should therefore be evaluated as a pathway from technical attribution to accountable clinical action (Figure 1).

### 2.11. Generalizability: From Multicentre Validation to Transportable Clinical Performance

Generalizability is not demonstrated by a large test set or by the inclusion of several hospitals in model development. A clinically transportable system should retain acceptable discrimination, calibration and decision performance when applied to patients, devices, institutions and time periods that were not represented during training. This distinction is particularly important in gastroenterology, where image appearance is influenced by endoscope manufacturer, acquisition settings, bowel preparation, operator technique, pathology processing and scanner protocols, while disease prevalence and clinical thresholds vary across healthcare settings. Multicentre development may increase heterogeneity, but it does not constitute external validation when data from all centres are pooled before random splitting or when the model is adjusted after exposure to the target cohort.

Direct evidence of dataset shift shows why internal performance can be misleading. A convolutional neural network for pancreatic cancer detection achieved areas under the receiver operating characteristic curve of 0.997 and 0.999 in two Taiwanese test sets, but this decreased to 0.920 in an independent US cohort. More importantly, sensitivity for tumours smaller than 2 cm fell from 92.1% locally to 63.1% in the US cohort [63]. Although overall discrimination remained potentially useful, the loss of sensitivity occurred in the subgroup for which diagnostic assistance may be most valuable. This illustrates why a modest change in an aggregate performance statistic can conceal a clinically consequential failure.

Hardware-related shift is especially relevant to endoscopic AI. In one study, an algorithm recognising the oesophagogastric junction achieved an accuracy exceeding 0.979 when evaluated using images acquired with the endoscope model represented during training, but its performance deteriorated on images from other endoscope models and manufacturers. Cycle-consistent domain adaptation produced statistically significant improvements, with increases in the area under the curve ranging from approximately 0.006 to 0.062 [64]. These findings confirm that models may learn manufacturer-specific colour, contrast and resolution characteristics alongside clinically relevant features. Domain adaptation can mitigate this problem, but it changes the original model and requires access to representative target-domain data. Performance after adaptation should therefore be reported as local recalibration or model updating rather than evidence that the unmodified system was intrinsically transportable.

Similar effects are evident outside endoscopy. A four-centre gastric tumour segmentation study found consistently different Dice similarity coefficients between within-centre and cross-centre testing, with the largest decline from 67.28% to 60.62%. Domain-adaptive training partially recovered performance to 62.20%, but did not eliminate the gap [65]. Variations in CT manufacturer, spatial resolution and reconstruction protocol were therefore not technical nuisances; they altered the model’s ability to define clinically relevant tumour boundaries. Adaptation methods are promising, but their evaluation should use locked test sets from genuinely unseen centres. Testing on a target domain that has already contributed unlabelled images during adaptation evaluates adaptation performance, not zero-shot generalizability.

Several gastroenterology studies nevertheless show that performance can be preserved under more demanding validation. A model for oesophageal cancer and precursor lesions was prospectively evaluated using 836 previously unseen images from five hospitals. External accuracy ranged from 90.0% to 95.8%, compared with an internal accuracy of 95.6% [66]. In gastrointestinal pathology, an AI system trained on gastric biopsy slides was tested using 3450 samples from 1772 patients across ten institutions and achieved a mean tissue-level accuracy of 94.6%, with relatively limited between-centre variation [67]. This design is particularly informative because staining, slide preparation and scanning practices can introduce substantial laboratory-specific variation.

Prospective video-level evaluation provides stronger evidence than validation on retrospectively selected still images. ENDOANGEL-CPS, developed to estimate colorectal polyp size, was evaluated using 157 prospective videos from three hospitals. Agreement with the reference measurement remained high, and the system produced fewer potentially inappropriate surveillance recommendations than participating endoscopists [68]. Similarly, an AI-assisted capsule endoscopy system was evaluated across European and US centres using two capsule platforms. In 259 examinations, AI-assisted reading achieved 90.2% sensitivity for ulcers and erosions, compared with 69.6% under standard-of-care reading, although specificity was lower at 84.4% versus 99.4% [69]. The latter finding demonstrates that transportability should not be reduced to sensitivity alone: the balance between missed lesions, false-positive findings and reading burden determines whether performance remains acceptable for the intended workflow.

Generalisability also concerns clinical populations rather than imaging technology alone. A mortality model developed in a prospective cohort of 7239 hospitalised patients with cirrhosis from 115 centres achieved an overall area under the curve of 0.815. Performance varied across economic settings, from 0.768 in low- and lower-middle-income countries to 0.867 in upper-middle-income countries. External validation in 28,670 US veterans produced an area under the curve of 0.859 [70]. This is unusually broad validation, but the external cohort was 96% male and differed substantially in age, disease severity and mortality prevalence. Preserved discrimination in this population does not remove the need to assess calibration and clinical thresholds in women, younger patients or health systems with different admission and treatment practices.

Transportability must also be judged against the intended role of the model. FIB-6 was initially derived using machine learning in patients with chronic hepatitis C and was subsequently evaluated in 2472 patients with metabolic dysfunction-associated fatty liver disease from six countries. Its high negative predictive value supported its proposed rule-out function, whereas comparatively low specificity limited its suitability as a definitive diagnostic tool [71]. Thus, a model can be transportable for excluding advanced disease without being sufficiently reliable for confirming it or initiating an invasive intervention.

Robust evaluation should therefore treat external validation as a structured stress test. A locked model should be assessed prospectively or temporally in centres that did not contribute to development, with results stratified by institution, device, acquisition protocol, geography, demographic group and disease spectrum. Discrimination should be accompanied by calibration-in-the-large, calibration slope, clinically relevant sensitivity and specificity, and decision-curve or net-benefit analyses. Performance at the intended decision threshold is more informative than an overall area under the curve. Recalibration, domain adaptation and threshold adjustment should be explicitly documented, version controlled and re-evaluated after implementation.

Finally, transportability is also a communication obligation. Clinicians should be informed about the populations, devices and settings in which a system was validated, the conditions under which its performance deteriorated, and whether local recalibration has been performed. When an estimate is communicated to a patient, uncertainty arising from limited population similarity should not be presented as if it were ordinary statistical uncertainty. A model becomes generalizable in the clinically relevant sense only when its performance, calibration and consequences remain acceptable in the setting where the decision is actually made.

### 2.12. Clinical Actionability: From Model Performance to Patient and System Benefit

Clinical actionability can be considered along a continuum from model performance to reader performance, changes in clinician behaviour and management, workflow effects, patient-important outcomes and, ultimately, health-system consequences. An AI system becomes clinically actionable when its output is delivered at a defined decision point, linked to a specified response and shown to improve care without disproportionate harm or resource use.

Randomised trials of computer-aided detection illustrate that technical efficacy does not translate uniformly into clinical benefit. In COLO-DETECT, a pragmatic trial involving 2032 participants across 12 National Health Service hospitals, AI-assisted colonoscopy increased mean adenomas per procedure from 1.21 to 1.56 and adenoma detection from 48.4% to 56.6%, without an apparent increase in serious adverse events [19]. A community-based pragmatic trial of 1100 patients similarly increased adenoma detection from 34.4% to 42.5%, although the gain was driven predominantly by lesions smaller than 5 mm [72]. These findings support CADe as an effective second observer in some routine-care environments.

However, benefits have not been consistent across settings. In AI-SEE, which enrolled 769 patients from four community endoscopy centres, CADe did not improve adenomas per colonoscopy or adenoma detection rate. It instead increased the removal of non-adenomatous, non-serrated polyps and prolonged mean withdrawal time by approximately one minute [73]. In AI-DETECT, conducted within an English bowel cancer screening programme, CADe produced a borderline improvement in overall polyp detection but no statistically significant improvement in adenoma detection among experienced, high-performing colonoscopists [74]. Collectively, these trials suggest that the incremental value of CADe depends on baseline operator performance, clinical indication, lesion spectrum, existing mucosal-exposure practices and the threshold at which alerts trigger resection. An increase in highlighted or removed lesions should not automatically be interpreted as better care.

Workflow effects can further modify efficacy. In the community-based pragmatic trial, the improvement in adenoma detection was greater during the first half of the study and was no longer statistically evident during the second half [72]. A subsequent analysis of 1700 colonoscopies found that CADe-assisted adenoma detection was higher during the first three procedures of the day but declined during later procedures, when its advantage disappeared. The authors attributed this pattern to possible alert fatigue and reduced responsiveness to repeated prompts [75]. Although observational, these findings show that an algorithm’s effect cannot be separated from the attention, workload and behaviour of its users.

Not every workflow intervention produces the intended behavioural change. An AI-assisted speed-monitoring system designed to discourage rapid colonoscope withdrawal was evaluated in a clinical implementation study involving 332 patients. Mean withdrawal time, the proportion of procedures meeting the six-minute threshold and adenoma detection were almost identical before and after implementation [76]. Providing feedback was therefore insufficient when baseline withdrawal performance was already satisfactory. By contrast, AI-assisted capsule endoscopy identified 94.7% of lesions established by expert review and reduced median reporting time to 172 s, although higher sensitivity was accompanied by lower specificity than standard reading [69]. Workflow evaluation must consequently consider not only time saved but also the downstream burden created by false-positive findings, confirmatory review and additional intervention.

Evidence that AI changes treatment pathways and patient outcomes remains limited. A notable implementation study used Danish registry data to develop an AI model estimating one-year mortality after colorectal cancer surgery and linked predicted risk groups to increasingly intensive perioperative interventions. Following implementation, the proportion of patients with a comprehensive complication index above 20 was 19.1%, compared with 28.0% in the preceding standard-care cohort; medical complications occurred in 23.7% and 37.3%, respectively [77]. This study moves beyond prediction by embedding model output within a predefined care pathway. Nevertheless, its non-randomised before-and-after design cannot isolate the contribution of the algorithm from that of the accompanying perioperative intervention bundle, temporal changes or differences between cohorts. The clinically evaluated intervention was the AI-informed pathway rather than the prediction model alone.

This distinction is fundamental. An actionable system must specify who receives the output, when it is presented, what action it recommends and who remains accountable. A probability estimate without a defined response is prognostic information, not decision support. Conversely, a care pathway may improve outcomes even when the model itself has only moderate discrimination, provided that its risk categories reliably direct effective interventions. Implementation studies should therefore evaluate the complete sociotechnical intervention: model, interface, users, clinical protocol, communication process and downstream care.

Health-economic analyses offer a broader assessment of value, but the available evidence is predominantly model-based. A US microsimulation estimated that AI-assisted screening colonoscopy could reduce colorectal cancer incidence and mortality while saving US$57 per screened individual. Extrapolation to the national level projected 7194 fewer colorectal cancer cases, 2089 fewer deaths and annual savings of US$290 million [78]. These estimates depended on the assumption that improvements in adenoma detection observed in trials would persist across repeated screening cycles and translate into long-term cancer prevention.

A separate microsimulation showed that CADe would be cost-effective if it increased mean adenoma detection from 26% to at least 30% or cost less than US$579 per colonoscopy [79]. This threshold-based finding is informative because it makes economic value conditional on locally realised clinical benefit. A system that produces little incremental improvement among high-performing endoscopists may not be cost-effective even if its acquisition cost is modest. Conversely, targeted deployment among lower-performing operators could provide greater value than universal adoption.

More conservative modelling has challenged assumptions of substantial population benefit. A European microsimulation estimated only small and uncertain reductions in ten-year colorectal cancer incidence with CADe. For primary colonoscopy screening, the estimated reduction was 0.11 percentage points, whereas the proportion undergoing surveillance colonoscopy increased by 6.37 percentage points. The certainty of all outcomes was rated as low [80]. Thus, improved detection may create additional surveillance, pathology and procedural demand without a proportionate reduction in cancer outcomes. These downstream consequences should be included in economic evaluations rather than treating every additional adenoma as an unqualified benefit.

Economic evidence outside colonoscopy is emerging. A model evaluating AI-enhanced MRI surveillance for hepatocellular carcinoma in Italian patients with cirrhosis estimated an incremental cost-effectiveness ratio of approximately €9888 per quality-adjusted life-year gained. The result was most sensitive to the cost, sensitivity and specificity of the AI system [81]. As with colonoscopy, this represents projected rather than observed value. Prospective studies that measure diagnostic stage shift, treatment eligibility, resource use and patient outcomes are needed before economic benefit can be established.

Future implementation research should use pragmatic, cluster-randomised or stepped-wedge designs where feasible and should distinguish algorithm efficacy from the effect of the associated care pathway. Relevant outcomes include changes in management, missed clinically important lesions, unnecessary biopsy or resection, procedure and reporting time, alert burden, clinician override, complications, patient-reported outcomes and total downstream costs. Performance should also be monitored after deployment because alert fatigue, changing case mix, software updates and evolving clinical practice may alter effectiveness over time.

Clinical actionability depends on linking model output to a defined and accountable clinical response, with uncertainty communicated at the point of decision. Representative studies demonstrate that explainability, external validity and clinical impact remain unevenly distributed, with few systems providing strong evidence across all three dimensions (Table 1).

Table 1 presents selected studies that exemplify variation in the maturity of evidence across explainability, generalisability and clinical actionability. Priority was given to studies providing prospective or multicentre evaluation, independent validation, human–AI assessment, clinically meaningful outcomes or informative negative findings.

### 2.13. Governance, Regulation and Human Oversight: Accountability Across the AI Lifecycle

Professional guidance increasingly converges on a central principle: artificial intelligence should augment clinical expertise rather than replace it. Early statements from the American Society for Gastrointestinal Endoscopy (ASGE) prioritised standardised datasets, rigorous validation and prospective studies measuring clinically meaningful outcomes [15]. The European Society of Gastrointestinal Endoscopy (ESGE) subsequently proposed task-specific performance thresholds linked to established quality indicators [82]. More recent frameworks have broadened this agenda to include workflow integration, cost, education, human–AI interaction and lifecycle governance [16,83,84]. These documents provide an important professional standard, but remain consensus-based guidance rather than binding regulation.

Regulatory authorisation and clinical value should also be distinguished. Market access establishes whether a system meets the applicable requirements for its stated intended use; it does not establish that the system improves patient-important outcomes, is cost-effective or will retain its performance in every clinical setting. This distinction is illustrated by the 2025 AGA living guideline on computer-aided detection: despite improved adenoma detection, the panel could not recommend for or against routine use because evidence for longer-term benefits, harms and resource consequences remained of very low certainty [85]. Governance should therefore begin with a clearly defined clinical purpose and an explicit account of the decision that the system is intended to change, not simply evidence that its discrimination exceeds a technical benchmark.

Professional consensus is increasingly moving in this direction. A recent American College of Gastroenterology Delphi process emphasised heterogeneous validation data, protection against deskilling, specialty-society oversight and payment models tied to quality and value [86]. The World Endoscopy Organization identified data governance, medicolegal responsibility, equity and bias as core requirements for responsible implementation [87]. Importantly, fairness cannot be inferred from aggregate accuracy. Differences in access to screening, disease prevalence, phenotype, image quality and documentation practices can all be reproduced or amplified by an algorithm, particularly when under-represented populations are absent from development and validation datasets [14].

The regulatory architecture is also shifting from one-time approval towards lifecycle oversight. In the European Union, AI systems that are medical-device products or safety components and require third-party conformity assessment can fall within the high-risk framework of the AI Act. The resulting requirements include risk management, data governance, technical documentation, traceability, appropriate human oversight, robustness, cybersecurity and post-market monitoring, operating alongside the existing medical-device framework [88]. In the United States, the Food and Drug Administration’s guidance on predetermined change-control plans provides a mechanism through which manufacturers can prospectively specify intended modifications, their validation procedures and their anticipated effects on safety and effectiveness. Both approaches recognise that an adaptive or periodically updated model cannot be governed as a static instrument.

### 2.14. Making Human Oversight Meaningful

Human oversight should not be reduced to placing a clinician nominally “in the loop”. It is meaningful only if the clinician understands the system’s intended use and failure modes, can inspect the underlying clinical information, has sufficient time and authority to reject its output, and can proceed safely when the system is unavailable. The World Health Organisation similarly positions human autonomy, transparency, accountability, inclusiveness and safety as foundational principles for healthcare AI [89].

Empirical evidence suggests that both patients and clinicians expect this supervisory role. In an intercontinental World Endoscopy Organization survey, 92.3% of patients considered endoscopist oversight necessary, whereas endoscopists expressed concerns about liability, procedural burden and dependence on AI [90]. However, assigning responsibility entirely to the endoscopist would be problematic when the clinician cannot inspect the training data, modify system updates or independently detect distributional drift. Responsibility should instead be distributed across the AI lifecycle: developers are responsible for defining the intended use, documenting limitations and validating updates; healthcare organisations are responsible for procurement, local evaluation, training, monitoring and incident response; and clinicians remain responsible for interpreting the output within the patient’s clinical context.

Training is consequently part of governance rather than an optional educational addition. The ESGE curriculum recommends baseline procedural competence, AI literacy, recognition of automation bias, avoidance of over-reliance and continuous monitoring of quality indicators [16]. Clinicians should be trained not only to operate a system but also to recognise discordance, for example, a CADe alert unsupported by the endoscopic appearance or a risk estimate generated from incomplete clinical data. Independent diagnostic and procedural competence must be preserved so that human oversight remains an effective safety mechanism rather than retrospective endorsement of an algorithmic recommendation.

### 2.15. From Deployment to Continuous Surveillance

Before deployment, institutions should specify the target population, clinical pathway, expected benefit, unacceptable failure modes and conditions under which the system should not be used. Regulatory clearance should be supplemented by local validation whenever the patient population, endoscopy platform, image-processing chain or clinical workflow differs materially from the development environment. Procurement decisions should also address data access, cybersecurity, software versioning, update policies, interoperability, auditability and the allocation of responsibility following an adverse event [83,84,87].

After deployment, monitoring should extend beyond aggregate accuracy. Relevant indicators include calibration, alert and override rates, procedure time, false-positive burden, downstream investigations, patient outcomes and performance across demographic, clinical and technical subgroups. A system may remain accurate overall while deteriorating in a specific hospital, device generation or patient group. Model updates should therefore be treated as governed change events, with version control, predefined validation criteria, documented approval and the capacity to suspend or roll back the system when safety signals emerge [88].

These safeguards are equally important for generative AI used in documentation, patient education, guideline retrieval or administrative decision support. Even where such tools are not regulated as medical devices, their outputs can influence clinical reasoning and communication. Institutional oversight should therefore define which data may be entered, how generated content is verified, whether AI involvement is recorded and when human review is mandatory [86,89].

Governance should also ensure proportionate disclosure when AI materially influences diagnosis, surveillance or treatment, including its role, residual uncertainty and the availability of human review [87,89,90].

**Figure 2:**
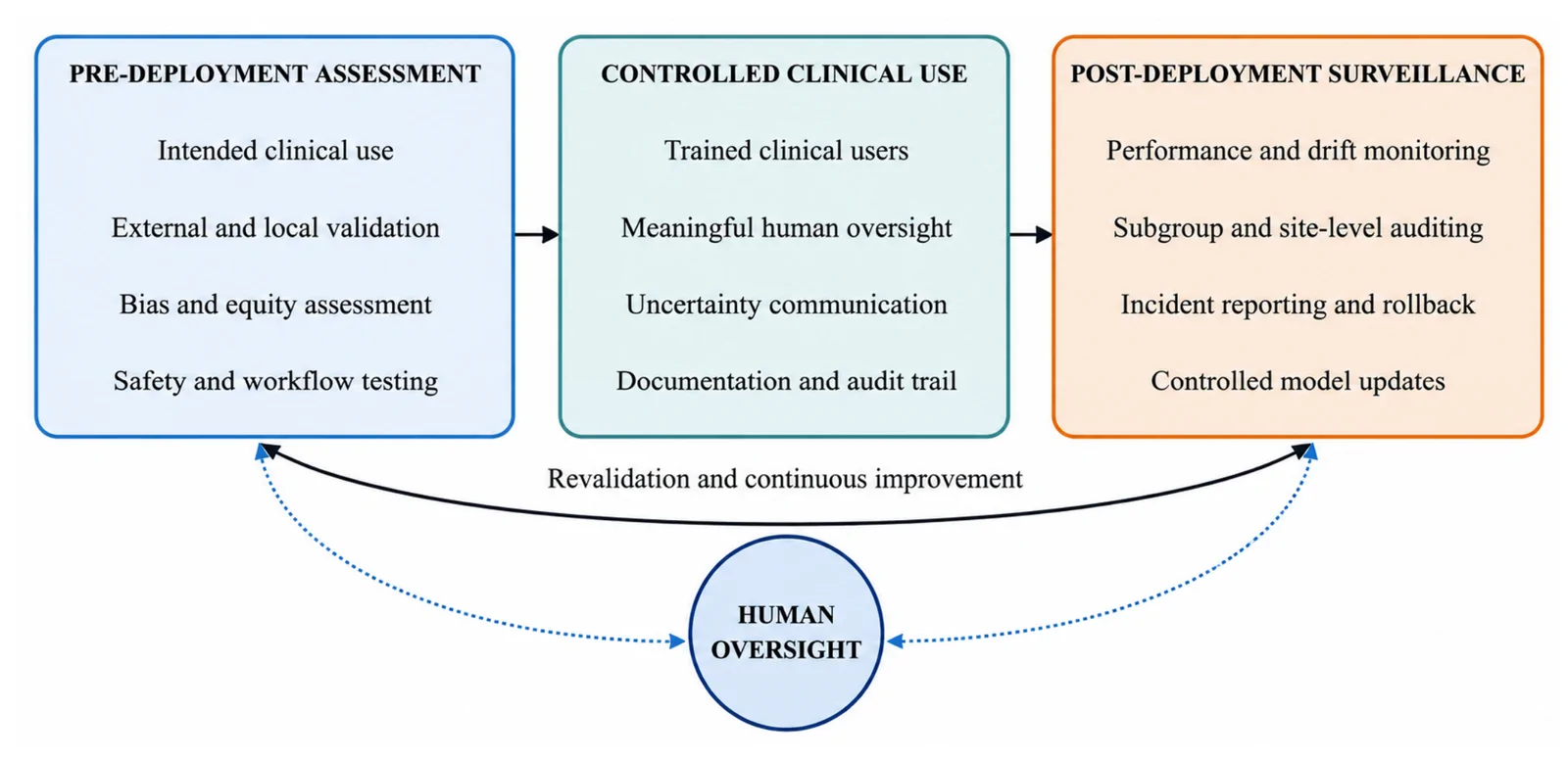
Lifecycle governance of clinical AI in gastroenterology. Governance spans pre-deployment assessment, controlled clinical use and post-deployment surveillance. These phases address intended use, local validity, human oversight, uncertainty communication, performance drift, subgroup disparities and model updates. Transparency, accountability, data governance, cybersecurity and health equity apply across the lifecycle, with responsibilities shared among developers, healthcare organisations, clinicians, patients and regulators. This conceptual framework was developed by the authors based on [15,16,83,84,86].

Taken together, regulatory approval, professional guidance and human oversight should be understood as complementary layers. Trustworthy AI in gastroenterology will depend less on declaring a system autonomous or explainable than on ensuring that its purpose, performance, limitations, ownership and consequences remain visible and that responsibility remains assignable throughout its clinical lifecycle. Accordingly, governance should be treated as a continuous clinical lifecycle rather than a one-time regulatory checkpoint, integrating pre-deployment assessment, meaningful human oversight and post-deployment surveillance (Figure 2).

### 2.16. Communication, Trust and Shared Decision Making: Translating AI Outputs into Care

Clinical actionability is mediated by communication. An AI output becomes useful only when clinicians can interpret its relevance, recognise its uncertainty and explain its implications to patients. Communication is therefore not a secondary interface issue; it determines whether an algorithm supports deliberation, introduces automation bias or merely adds another signal to an already complex workflow.

Trust should not be treated as an unqualified objective. The relevant goal is *calibrated trust*: reliance when the system is likely to be correct and scepticism when its output conflicts with clinical evidence. Clinically grounded explanations improved acceptance in gastric and pancreatic lesion assessment [9,61], but explanation alone does not ensure appropriate reliance. In optical diagnosis of colorectal polyps, displaying CADx predictions increased accuracy from 69.3% to 76.6%, although use of the prediction depended on how confidence information was presented and whether consultation was optional or mandatory [91]. Similarly, a randomised video-based Barrett’s oesophagus study found both algorithm aversion—correct AI guidance was disregarded in 16% of cases—and over-reliance, with 9% of initially correct assessments changed to incorrect after AI assistance [92]. Together with evidence of alert fatigue during repeated CADe use [75] these findings show that human–AI performance cannot be inferred from standalone model accuracy.

Attitudinal evidence indicates broad but conditional acceptance. A systematic review of 13 studies involving 1538 providers found that 80% were interested in using AI-assisted colonoscopy and 74% expected it to improve detection, while cost and accountability remained prominent concerns [12]. Patients express a similar conditional position: most support AI-assisted image analysis, but strongly prefer continued endoscopist oversight [90]. Acceptability also varies with age, education, familiarity with AI and perceived effectiveness [93]. Implementation strategies should therefore avoid assuming either uniform enthusiasm or uniform resistance. Training should explicitly address failure modes, confidence interpretation, automation bias and the clinician’s authority to override the system [16,86].

Patient-facing large language models create a different communication challenge. A systematic review of medical chatbots in digestive diseases identified considerable heterogeneity in accuracy, completeness and safety evaluation [94]. Simplification can improve accessibility, but may also alter risk perception. In one study, 83% of patients considered AI-generated summaries of pathology reports beneficial, whereas pathologists identified omissions in 36% and potentially misleading content in 26% of evaluations; AI also assigned a substantially higher level of concern than the specialists [95]. Evaluations in coeliac disease reported misinformation in 13–24% of responses [96], while answers to irritable bowel syndrome questions were generally accurate but written at a reading level that could limit accessibility [97]. Expert-rated accuracy should therefore not be equated with patient comprehension or safe independent use. Patient-facing evidence remains comparatively sparse. Existing studies primarily address acceptability, perceived usefulness and the quality of AI-generated information, whereas the effects of AI on informed consent, understanding of uncertainty, calibration of trust and shared decision making have rarely been examined directly. These domains therefore remain important targets for prospective, patient-centred evaluation.

Shared decision making requires more than disclosing that AI was involved. Patients should understand the role of the system, the uncertainty surrounding its output, the available alternatives and whether the recommendation was independently reviewed by a clinician. This is particularly important when AI changes surveillance intervals, biopsy decisions or treatment selection. Although AI has been proposed as a means of supporting personalised decisions in inflammatory bowel disease [98], empirical evidence that it improves patient understanding, decisional conflict or concordance with patient preferences remains sparse. Future studies should evaluate comprehension, trust calibration, anxiety, decisional conflict, adherence and decision regret, while assessing whether communication performance differs across language and health-literacy groups.

Ultimately, effective communication should make AI-supported decisions intelligible and contestable. The clinician remains responsible for integrating the output with the patient’s history, values and preferences, while the patient should retain a meaningful opportunity to ask questions and request human review. Under these conditions, AI can support shared deliberation; without them, even a technically accurate system may weaken rather than strengthen patient-centred care.

### 2.17. A Translational Research Agenda for Trustworthy Clinical AI

The principal challenge for artificial intelligence in gastroenterology is no longer demonstrating that algorithms can recognise patterns. It is establishing when those patterns improve care. Translation should therefore follow a staged pathway in which clinical purpose, transportability, human–AI interaction, patient benefit and lifecycle governance are evaluated separately but connected prospectively.

First, development should begin with a clearly specified clinical use case. Investigators should define the target population, intended user, timing of the output, decision to be supported and action expected to follow. Predicting a diagnosis or outcome is insufficient when no effective response is available. New systems should also be compared with the existing care pathway rather than with an artificial benchmark or clinician working without routinely available information. Discrimination should be accompanied by calibration, clinically meaningful operating thresholds and decision-analytic measures that quantify the consequences of false-positive and false-negative results.

Second, external validation should become an entry requirement for clinical evaluation rather than a final addition to model development. The evidence reviewed here shows that apparently strong performance can deteriorate across institutions, populations and equipment platforms [64,65,66,67,68,69,70,71,72]. Validation cohorts should therefore differ meaningfully from development data in geography, time, clinical setting or technology. Results should be reported by site and relevant demographic, technical and disease subgroups rather than only as pooled averages. Prospective temporal validation using a locked model is particularly important because it approximates the conditions under which the system will initially encounter changing case mix and clinical practice.

Third, early clinical evaluation should examine the complete human–AI interaction. A silent deployment phase can identify integration failures, missing inputs and distributional drift before outputs are shown to clinicians. This should be followed by small-scale live evaluation of usability, workload, alert burden, clinician override and the consequences of incorrect or discordant recommendations. DECIDE-AI provides an appropriate framework for reporting these early-stage evaluations, including the clinical setting, users, workflow and error cases [99]. Human-centred assessment should determine whether explanations improve appropriate reliance, not merely whether they increase confidence and whether uncertainty can be communicated to patients without distorting risk perception [12,91,92,93,94,95,96,97,98].

Fourth, prospective impact studies should evaluate the AI-supported pathway rather than the algorithm in isolation. Pragmatic, cluster-randomised or stepped-wedge designs may be more appropriate than individually randomised explanatory trials when the intervention changes workflow or operator behaviour. Outcomes should include clinically important missed lesions, unnecessary biopsy or resection, treatment modification, complications, procedure and reporting time, patient-reported outcomes, disparities and total downstream costs. CONSORT-AI and SPIRIT-AI require explicit reporting of the AI intervention, input–output handling, user expertise, integration setting, human–AI interaction and error analysis [100,101]. These frameworks improve transparency but do not substitute for representative data, suitable comparators or patient-important endpoints.

Finally, evaluation must continue after deployment. Monitoring should track calibration, failure and override rates, subgroup performance, workflow disruption and safety incidents. Software versions and model updates should be documented, and material changes should trigger predefined revalidation and, where necessary, suspension or rollback [88]. TRIPOD+AI provides harmonised reporting guidance for prediction-model studies using regression or machine-learning methods and should improve the reproducibility and appraisal of both development and validation evidence [102]. Institutions should additionally define responsibility for monitoring, incident investigation, patient communication and decisions to continue or discontinue use.

A trustworthy system is therefore not one that performs well once, but one that remains clinically useful across settings, users and time. Future research should measure success by whether AI produces better, more equitable and more comprehensible decisions under real clinical conditions, not by whether it achieves another incremental improvement in retrospective accuracy.

## 3. Conclusions

Artificial intelligence is beginning to influence gastroenterology across endoscopic detection and characterisation, inflammatory bowel disease assessment, hepatology, and pancreaticobiliary decision support. The most mature evidence is found in gastrointestinal endoscopy, particularly computer-aided polyp detection, where randomised and pragmatic studies have demonstrated improvements in selected process-level outcomes. However, these gains have not been consistent across clinical settings and have not yet established proportional improvements in patient-important outcomes. Evidence in other subspecialties remains dominated by retrospective development and validation studies.

The central translational challenge is therefore not algorithmic performance alone, but the alignment of explainability, generalizability and clinical actionability. Current explanations are largely technical and are rarely evaluated for their effects on clinician understanding, trust calibration or patient communication. External validation is increasing, but geographic, technological and demographic transportability, calibration and performance under dataset shift remain insufficiently examined. Prospective implementation studies, health-economic evaluations and analyses of downstream harms are comparatively uncommon.

Clinical AI should consequently be evaluated as part of a sociotechnical intervention comprising the model, interface, users, workflow, communication process and associated care pathway. Meaningful human oversight requires clinicians who understand system limitations, can reject discordant outputs and retain sufficient independent expertise to avoid automation dependence. Patients should be informed when AI materially influences diagnosis, surveillance or treatment, with uncertainty and alternatives communicated in an accessible form that supports shared decision making.

Future studies should prioritise clinically defined use cases, representative multicentre validation, prospective evaluation and continuous post-deployment surveillance. Success should be measured through changes in management, patient outcomes, unnecessary interventions, workflow, equity and cost not solely through retrospective discrimination. AI is most likely to contribute to gastroenterology when it functions as transparent, contestable and continuously monitored decision support. Its value will ultimately depend not on whether it can reproduce clinical judgements, but on whether it helps clinicians and patients make safer, more equitable and better-informed decisions. Overall, these findings highlight the growing clinical utility of AI and its potential to complement, rather than to replace, clinical expertise.

## Figures and Tables

**Table 1:** Representative evidence for explainability, generalizability and clinical actionability of artificial intelligence in gastroenterology.

Study and Clinical Task	Design and Setting	Explainability	Generalisability	Clinical or Workflow Effect	Key Limitation
Dong et al.; early gastric neoplasia under white-light endoscopy [9]	4482 images and 296 videos from 3279 patients across eight hospitals; multi-reader study involving 77 endoscopists	Clinically grounded features; trust and acceptance were directly measured	Internal, external and consecutive video testing	AI assistance increased endoscopist accuracy from 70.61% to 79.63%; explainable AI received higher trust and acceptance scores than black-box AI	Controlled reader evaluation; effect on routine behaviour and patient outcomes was not assessed
Cui et al.; EUS diagnosis of solid pancreatic lesions [61]	Four-centre randomised crossover study; 628 retrospective and 130 prospective patients; 12 endoscopists	Multimodal interpretability information was presented to users	Three independent external cohorts plus prospective evaluation	Novice accuracy increased from 0.69 to 0.90; interpretability information reduced scepticism among experienced endoscopists	Short-term reader performance; limited number of endoscopists and centres within one country
Thijssen et al.; textual description of colorectal polyp features [59]	Development study involving 55 colorectal polyps and expert reference descriptions	Automatically generated clinically recognisable textual features	No independent clinical deployment cohort	Agreement with expert descriptions ranged from 0.167 for pit distribution to 0.957 for pit type	Small dataset; explanation quality varied substantially across features; decision impact was not evaluated
Graham et al.; screening of large-bowel biopsies [54]	6591 whole-slide images from 3291 patients; one development and three external institutions	Heatmaps and quantitative histological features	Three independent external datasets; mean AUC approximately 0.97	At 99% sensitivity, the model was estimated to reduce normal slides requiring review by approximately 55%	Workload reduction was estimated rather than prospectively observed
Park et al.; identification of the oesophagogastric junction [64]	45,683 images from 1498 examinations; validation in 624 separate cases using different endoscope systems	Technical image transformation through CycleGAN	Explicit cross-device and cross-manufacturer evaluation	Performance deteriorated on untrained endoscope models; domain adaptation increased AUC by approximately 0.006–0.062	Adaptation required target-domain data and produced a modified rather than intrinsically transportable model
Liu et al.; pancreatic cancer detection on CT [63]	Taiwanese development cohorts with independent US validation	No human-centred explanation evaluation	Geographic and cross-racial external validation	AUC decreased from 0.997 to 0.999 locally to 0.920 in the US cohort; sensitivity for tumours smaller than 2 cm decreased from 92.1% to 63.1%	Retrospective design; clinically important subgroup deterioration was obscured by acceptable overall AUC
Abe et al.; AI-assisted diagnosis of gastric biopsies [67]	Training on 4511 biopsy tissues; validation using 3450 samples from 1772 patients across ten institutions	Histological feature distribution incorporated into model architecture; no user study	Multi-institutional pathology validation	Mean tissue-level accuracy was 94.6% with limited between-centre variation	Retrospective slide evaluation; effects on pathologist workload and diagnostic errors were not prospectively measured
Andrade et al.; ulcers and erosions in Crohn’s disease capsule endoscopy [69]	259 examinations from European and US centres using two capsule platforms	Lesion identification supplied to the reader; explanation comprehension was not assessed	Multicentre, international and cross-device validation	AI-assisted sensitivity was 90.2% versus 69.6% for standard reading; median reporting time was 172 s	Specificity was lower with AI assistance (84.4% versus 99.4%); downstream consequences of false positives were not measured
Silvey et al.; inpatient mortality in cirrhosis [70]	Prospective cohort of 7239 patients from 115 international centres; external validation in 28,670 US veterans	Variable importance reported; no clinician-facing explanation evaluation	Global development, income-stratified analysis and independent external validation	AUC was 0.815 in the global cohort and 0.859 in the external cohort	Performance varied by country-income group; the external cohort was 96% male and differed substantially in case mix
Seager et al.; CADe-assisted colonoscopy in COLO-DETECT [19]	Pragmatic randomised trial involving 2032 participants across 12 NHS hospitals	Real-time visual alerts; explanation was not separately evaluated	Multicentre routine-practice evaluation	Mean adenomas per procedure increased from 1.21 to 1.56 and ADR from 48.4% to 56.6%	No evidence yet that additional detection reduces long-term colorectal cancer incidence or mortality
Wei et al.; CADe in community colonoscopy, AI-SEE [73]	Multicentre randomised trial of 769 patients across four community centres	Real-time alerts without uncertainty communication	Community-based evaluation outside academic centres	No improvement in adenomas per colonoscopy or ADR; non-adenomatous polyp removal increased and withdrawal time was approximately one minute longer	Demonstrates potential burden without corresponding adenoma benefit; applicability may be system- and operator-dependent
Huang et al.; CADe alert fatigue [75]	Secondary analysis of a prospective real-world cohort containing 1700 colonoscopies	Repeated alerts evaluated as a human-factors exposure	Routine clinical implementation	CADe-associated ADR benefit was present during early procedures but disappeared later in the working day	Alert fatigue was inferred from temporal patterns rather than directly measured experimentally
Rosen et al.; perioperative decision support for colorectal cancer surgery [77]	National registry development involving 18,403 patients; local external validation and prospective implementation	Risk groups were linked to predefined perioperative intervention pathways	Registry-based validation followed by local clinical deployment	Severe postoperative complications decreased from 28.0% to 19.1%; any medical complication decreased from 37.3% to 23.7%	Non-randomised before-and-after design; the effect of AI cannot be separated from the accompanying intervention bundle
Areia et al. and Halvorsen et al.; CADe in colorectal cancer screening [78,80]	Markov and microsimulation models of population-based screening	Not applicable	Economic assumptions derived from selected trial and screening data	One model projected savings of US$57 per screened individual [78]; another estimated small, uncertain cancer benefit and a 6.37-percentage-point increase in surveillance colonoscopy [80]	Economic findings were simulated rather than observed and were highly dependent on assumed ADR improvement, surveillance rules and long-term cancer effects

Abbreviations: ADR, adenoma detection rate; AI, artificial intelligence; AUC, area under the receiver operating characteristic curve; CADe, computer-aided detection; CT, computed tomography; EUS, endoscopic ultrasonography; NHS, National Health Service. Notes: “Human-centred explainability” indicates that an explanation was evaluated with intended users rather than inferred from the presence of a heatmap or feature-importance method. “External validation” indicates testing in an institutionally, geographically or technologically independent cohort. The health-economic estimates in [78,80] are modelling results and should not be interpreted as observed cost savings.

## Data Availability

No new data were created or analyzed in this study. Data sharing is not applicable to this article.
